# Ethyl 2-(2-amino-5-methyl-1,2,4-triazolo[1,5-*a*]pyrimidin-7-yl)acetate

**DOI:** 10.1107/S1600536811054468

**Published:** 2011-12-23

**Authors:** Hicham Gueddar, Rachid Bouhfid, Ahmed Radouane Guessous, El Mokhtar Essassi, Seik Weng Ng

**Affiliations:** a Laboratoire de Chimie Organique Hétérocyclique, Pôle de Compétences Pharmacochimie, Université Mohammed V-Agdal, BP 1014 Avenue Ibn Batout, Rabat, Morocco; b Institute of Nanomaterials and Nanotechnology, MAScIR, Avenue de l’Armée Royale, Rabat, Morocco; c Department of Chemistry, University of Malaya, 50603 Kuala Lumpur, Malaysia; d Chemistry Department, King Abdulaziz University, PO Box 80203 Jeddah, Saudi Arabia

## Abstract

The nine-membered fused-ring of the title compound, C_10_H_13_N_5_O_2_, is approximately planar [maximum deviation = 0.012 (1) Å]; the bond angle at the methylene C atom is 111.33 (10)°. In the crystal, the amino group forms hydrogen bonds to the N atoms of the triazole rings of adjacent mol­ecules, generating a ribbon running along the *a* axis.

## Related literature

For a related mol­ecule, see: Fettouhi *et al.* (1996 ▶).
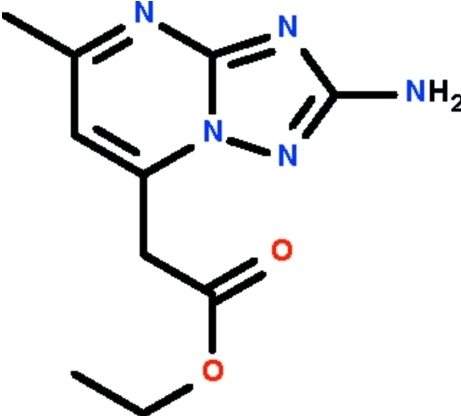


## Experimental

### 

#### Crystal data

C_10_H_13_N_5_O_2_
*M* *_r_* = 235.25
Monoclinic, 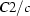

*a* = 22.9635 (4) Å
*b* = 7.7447 (1) Å
*c* = 14.7017 (3) Å
β = 124.574 (1)°
*V* = 2152.87 (6) Å^3^
*Z* = 8
Mo *K*α radiation
μ = 0.11 mm^−1^
*T* = 293 K
0.32 × 0.21 × 0.20 mm

#### Data collection

Bruker APEX DUO diffractometer
21320 measured reflections
4613 independent reflections
3058 reflections with *I* > 2σ(*I*)
*R*_int_ = 0.050

#### Refinement

*R*[*F* ^2^ > 2σ(*F* ^2^)] = 0.050
*wR*(*F* ^2^) = 0.138
*S* = 1.02
4613 reflections
163 parameters
2 restraints
H atoms treated by a mixture of independent and constrained refinement
Δρ_max_ = 0.50 e Å^−3^
Δρ_min_ = −0.32 e Å^−3^

### 

Data collection: *APEX2* (Bruker, 2010 ▶); cell refinement: *SAINT* (Bruker, 2010 ▶); data reduction: *SAINT*; program(s) used to solve structure: *SHELXS97* (Sheldrick, 2008 ▶); program(s) used to refine structure: *SHELXL97* (Sheldrick, 2008 ▶); molecular graphics: *X-SEED* (Barbour, 2001 ▶); software used to prepare material for publication: *publCIF* (Westrip, 2010 ▶).

## Supplementary Material

Crystal structure: contains datablock(s) global, I. DOI: 10.1107/S1600536811054468/xu5412sup1.cif

Structure factors: contains datablock(s) I. DOI: 10.1107/S1600536811054468/xu5412Isup2.hkl

Supplementary material file. DOI: 10.1107/S1600536811054468/xu5412Isup3.cml

Additional supplementary materials:  crystallographic information; 3D view; checkCIF report

## Figures and Tables

**Table 1:** Hydrogen-bond geometry (Å, °)

*D*—H⋯*A*	*D*—H	H⋯*A*	*D*⋯*A*	*D*—H⋯*A*
N5—H1⋯N2^i^	0.88 (1)	2.24 (1)	3.095 (1)	166 (2)
N5—H2⋯N3^ii^	0.89 (1)	2.15 (1)	3.037 (2)	175 (2)

Symmetry codes: (i) 
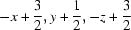
; (ii) 
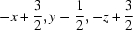
.

**Fig. 1.**
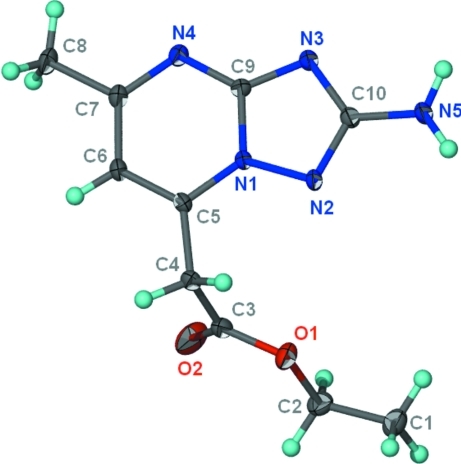
Thermal ellipsoid plot (Barbour, 2001) of C10H13N5O2 at the 70% probability level; hydrogen atoms are drawn as spheres of arbitrary radius.

## supplementary crystallographic information

### Comment

We reported the reaction of 3-diamino-1,2,4-triazole and 4-hydroxy-6-methyl-pyran-2-one to form ethyl 2-(2-amino-5-methyl-[1,2,4]triazolo[1,5-*a*]pyrimidin-7-yl)acetate (Fettouhi *et al.*, 1996), which is a member of a class of antiviral compounds. The use of 3,5-diamino-1,2,4-triazol with the pyrone gave the analogous amino-substituted compound (Scheme I). The nine-membered fused-ring of C_10_H_13_N_5_O_2_ is planar; the methylene unit connecting the fused-ring and the ethoxycarbonyl unit is slightly opened up to 111.33 (10)° (Fig. 1). The amino group forms hydrogen bonds to the N atoms of the triazole rings of adjacent molecules by a two-fold symmetry operation to generate a ribbon running along the *a*-axis of the monoclinic unit cell (Table 1).

### Experimental

A solution of 3,5-diamino-1,2,4-triazole (1 g, 10 mmol) and 4-hydroxy-6-methyl-pyran-2-one (1.6g, 12.6 mmol) in ethanol (30 ml) was heated for 12 hours. The solvent was removed by evaporation and the residue recrystallized from ethanol to afford the colorless crystals.

### Refinement

Carbon-bound H-atoms were placed in calculated positions (C–H 0.93–0.97 Å) and were included in the refinement in the riding model approximation, with *U*(H) set to 1.2–1.5*U*(C).

The amino H-atoms were located in a difference Fourier map, and were refined with a distance restraint of N–H 0.88±0.01 Å; their temperature factors were refined.

### Crystal data

**Table:**

C_10_H_13_N_5_O_2_	*F*(000) = 992
*M**_r_* = 235.25	*D*_x_ = 1.452 Mg m^−^^3^
Monoclinic, *C*2/*c*	Mo *K*α radiation, λ = 0.71073 Å
Hall symbol: -C 2yc	Cell parameters from 3608 reflections
*a* = 22.9635 (4) Å	θ = 2.8–35.5°
*b* = 7.7447 (1) Å	µ = 0.11 mm^−^^1^
*c* = 14.7017 (3) Å	*T* = 293 K
β = 124.574 (1)°	Prism, colorless
*V* = 2152.87 (6) Å^3^	0.32 × 0.21 × 0.20 mm
*Z* = 8	

### Data collection

**Table:**

Bruker APEX DUO diffractometer	3058 reflections with *I* > 2σ(*I*)
Radiation source: fine-focus sealed tube	*R*_int_ = 0.050
graphite	θ_max_ = 34.7°, θ_min_ = 2.8°
ω scans	*h* = −35→36
21320 measured reflections	*k* = −12→9
4613 independent reflections	*l* = −23→22

### Refinement

**Table:**

Refinement on *F*^2^	Primary atom site location: structure-invariant direct methods
Least-squares matrix: full	Secondary atom site location: difference Fourier map
*R*[*F*^2^ > 2σ(*F*^2^)] = 0.050	Hydrogen site location: inferred from neighbouring sites
*wR*(*F*^2^) = 0.138	H atoms treated by a mixture of independent and constrained refinement
*S* = 1.02	*w* = 1/[σ^2^(*F*_o_^2^) + (0.0666*P*)^2^ + 0.6364*P*] where *P* = (*F*_o_^2^ + 2*F*_c_^2^)/3
4613 reflections	(Δ/σ)_max_ = 0.001
163 parameters	Δρ_max_ = 0.50 e Å^−^^3^
2 restraints	Δρ_min_ = −0.32 e Å^−^^3^

### Fractional atomic coordinates and isotropic or equivalent isotropic displacement parameters (Å^2^)

**Table:**

	*x*	*y*	*z*	*U*_iso_*/*U*_eq_	
O1	0.66634 (4)	0.58516 (12)	0.48590 (7)	0.01669 (18)	
O2	0.59517 (5)	0.70201 (15)	0.31780 (7)	0.0279 (2)	
N1	0.60826 (5)	0.99885 (13)	0.53229 (7)	0.01076 (18)	
N2	0.67164 (5)	0.93485 (13)	0.61966 (7)	0.01190 (18)	
N3	0.66734 (5)	1.23064 (13)	0.63055 (8)	0.01260 (19)	
N4	0.55068 (5)	1.27125 (14)	0.46404 (8)	0.01363 (19)	
N5	0.77145 (5)	1.08068 (14)	0.76545 (8)	0.0147 (2)	
C1	0.77877 (6)	0.45604 (19)	0.56140 (11)	0.0200 (3)	
H1A	0.8124	0.4183	0.5466	0.030*	
H1B	0.7646	0.3596	0.5856	0.030*	
H1C	0.8000	0.5427	0.6181	0.030*	
C2	0.71486 (6)	0.53072 (18)	0.45759 (10)	0.0177 (2)	
H2A	0.7284	0.6284	0.4321	0.021*	
H2B	0.6928	0.4445	0.3995	0.021*	
C3	0.60866 (6)	0.66849 (16)	0.40788 (9)	0.0151 (2)	
C4	0.56130 (6)	0.71606 (16)	0.44533 (9)	0.0140 (2)	
H4A	0.5150	0.6649	0.3955	0.017*	
H4B	0.5811	0.6694	0.5186	0.017*	
C5	0.55386 (5)	0.90755 (15)	0.44764 (9)	0.0118 (2)	
C6	0.49644 (6)	1.00289 (16)	0.36969 (9)	0.0133 (2)	
H6	0.4573	0.9479	0.3099	0.016*	
C7	0.49650 (6)	1.18385 (16)	0.37998 (9)	0.0135 (2)	
C8	0.43393 (6)	1.28736 (18)	0.29316 (10)	0.0188 (2)	
H8A	0.4375	1.4029	0.3195	0.028*	
H8B	0.3912	1.2349	0.2773	0.028*	
H8C	0.4331	1.2904	0.2271	0.028*	
C9	0.60642 (6)	1.17669 (15)	0.54011 (9)	0.0114 (2)	
C10	0.70439 (6)	1.08132 (15)	0.67461 (9)	0.0115 (2)	
H1	0.7866 (9)	1.1735 (16)	0.8069 (12)	0.027 (4)*	
H2	0.7920 (8)	0.9812 (15)	0.7978 (13)	0.027 (4)*	

### Atomic displacement parameters (Å^2^)

**Table:**

	*U*^11^	*U*^22^	*U*^33^	*U*^12^	*U*^13^	*U*^23^
O1	0.0130 (4)	0.0209 (5)	0.0142 (4)	0.0049 (3)	0.0066 (3)	0.0026 (3)
O2	0.0328 (5)	0.0336 (6)	0.0165 (4)	0.0168 (5)	0.0135 (4)	0.0082 (4)
N1	0.0087 (4)	0.0096 (4)	0.0101 (4)	0.0000 (3)	0.0030 (3)	−0.0006 (3)
N2	0.0087 (4)	0.0104 (5)	0.0101 (4)	0.0000 (3)	0.0015 (3)	0.0003 (3)
N3	0.0108 (4)	0.0102 (4)	0.0118 (4)	−0.0001 (3)	0.0034 (3)	−0.0002 (3)
N4	0.0108 (4)	0.0114 (5)	0.0145 (4)	0.0010 (3)	0.0047 (3)	0.0007 (4)
N5	0.0122 (4)	0.0110 (5)	0.0113 (4)	−0.0002 (4)	0.0009 (3)	−0.0004 (4)
C1	0.0147 (5)	0.0207 (6)	0.0225 (6)	0.0038 (4)	0.0094 (4)	0.0025 (5)
C2	0.0178 (5)	0.0181 (6)	0.0180 (5)	0.0034 (5)	0.0106 (4)	−0.0006 (5)
C3	0.0148 (5)	0.0116 (5)	0.0138 (5)	0.0009 (4)	0.0050 (4)	−0.0017 (4)
C4	0.0118 (4)	0.0102 (5)	0.0153 (5)	−0.0013 (4)	0.0049 (4)	−0.0020 (4)
C5	0.0093 (4)	0.0116 (5)	0.0116 (4)	−0.0015 (4)	0.0043 (4)	−0.0025 (4)
C6	0.0091 (4)	0.0140 (5)	0.0115 (4)	−0.0001 (4)	0.0028 (4)	−0.0016 (4)
C7	0.0100 (4)	0.0147 (6)	0.0125 (4)	0.0007 (4)	0.0044 (4)	0.0003 (4)
C8	0.0122 (5)	0.0170 (6)	0.0178 (5)	0.0039 (4)	0.0028 (4)	0.0027 (5)
C9	0.0113 (4)	0.0087 (5)	0.0124 (4)	0.0004 (4)	0.0057 (4)	0.0000 (4)
C10	0.0112 (4)	0.0107 (5)	0.0104 (4)	−0.0007 (4)	0.0048 (4)	0.0001 (4)

### Geometric parameters (Å, °)

**Table:**

O1—C3	1.3276 (14)	C1—H1B	0.9600
O1—C2	1.4555 (15)	C1—H1C	0.9600
O2—C3	1.2049 (15)	C2—H2A	0.9700
N1—C5	1.3582 (14)	C2—H2B	0.9700
N1—N2	1.3755 (12)	C3—C4	1.5169 (17)
N1—C9	1.3847 (15)	C4—C5	1.4954 (17)
N2—C10	1.3474 (15)	C4—H4A	0.9700
N3—C9	1.3374 (14)	C4—H4B	0.9700
N3—C10	1.3621 (15)	C5—C6	1.3720 (15)
N4—C7	1.3373 (14)	C6—C7	1.4096 (17)
N4—C9	1.3419 (14)	C6—H6	0.9300
N5—C10	1.3494 (14)	C7—C8	1.5016 (16)
N5—H1	0.877 (9)	C8—H8A	0.9600
N5—H2	0.887 (9)	C8—H8B	0.9600
C1—C2	1.5085 (17)	C8—H8C	0.9600
C1—H1A	0.9600		
			
C3—O1—C2	116.37 (9)	C5—C4—H4A	109.4
C5—N1—N2	127.13 (10)	C3—C4—H4A	109.4
C5—N1—C9	122.57 (9)	C5—C4—H4B	109.4
N2—N1—C9	110.30 (9)	C3—C4—H4B	109.4
C10—N2—N1	101.03 (9)	H4A—C4—H4B	108.0
C9—N3—C10	103.08 (10)	N1—C5—C6	115.69 (11)
C7—N4—C9	116.18 (10)	N1—C5—C4	118.66 (9)
C10—N5—H1	117.7 (11)	C6—C5—C4	125.64 (10)
C10—N5—H2	119.6 (11)	C5—C6—C7	120.23 (10)
H1—N5—H2	117.3 (15)	C5—C6—H6	119.9
C2—C1—H1A	109.5	C7—C6—H6	119.9
C2—C1—H1B	109.5	N4—C7—C6	123.11 (10)
H1A—C1—H1B	109.5	N4—C7—C8	117.06 (11)
C2—C1—H1C	109.5	C6—C7—C8	119.83 (10)
H1A—C1—H1C	109.5	C7—C8—H8A	109.5
H1B—C1—H1C	109.5	C7—C8—H8B	109.5
O1—C2—C1	106.55 (10)	H8A—C8—H8B	109.5
O1—C2—H2A	110.4	C7—C8—H8C	109.5
C1—C2—H2A	110.4	H8A—C8—H8C	109.5
O1—C2—H2B	110.4	H8B—C8—H8C	109.5
C1—C2—H2B	110.4	N3—C9—N4	128.53 (11)
H2A—C2—H2B	108.6	N3—C9—N1	109.22 (9)
O2—C3—O1	124.23 (11)	N4—C9—N1	122.23 (10)
O2—C3—C4	123.83 (11)	N2—C10—N5	121.70 (10)
O1—C3—C4	111.95 (10)	N2—C10—N3	116.37 (9)
C5—C4—C3	111.33 (10)	N5—C10—N3	121.86 (10)
			
C5—N1—N2—C10	178.81 (11)	C9—N4—C7—C8	−179.26 (11)
C9—N1—N2—C10	−0.44 (12)	C5—C6—C7—N4	−0.35 (18)
C3—O1—C2—C1	174.51 (11)	C5—C6—C7—C8	178.90 (11)
C2—O1—C3—O2	−0.81 (19)	C10—N3—C9—N4	−178.56 (12)
C2—O1—C3—C4	178.92 (10)	C10—N3—C9—N1	0.13 (12)
O2—C3—C4—C5	−62.89 (16)	C7—N4—C9—N3	178.85 (11)
O1—C3—C4—C5	117.38 (11)	C7—N4—C9—N1	0.31 (16)
N2—N1—C5—C6	−179.22 (10)	C5—N1—C9—N3	−179.09 (10)
C9—N1—C5—C6	−0.05 (16)	N2—N1—C9—N3	0.20 (13)
N2—N1—C5—C4	−0.24 (17)	C5—N1—C9—N4	−0.30 (17)
C9—N1—C5—C4	178.93 (10)	N2—N1—C9—N4	178.99 (10)
C3—C4—C5—N1	−76.46 (13)	N1—N2—C10—N5	−176.45 (10)
C3—C4—C5—C6	102.41 (13)	N1—N2—C10—N3	0.56 (13)
N1—C5—C6—C7	0.35 (16)	C9—N3—C10—N2	−0.45 (13)
C4—C5—C6—C7	−178.55 (11)	C9—N3—C10—N5	176.55 (10)
C9—N4—C7—C6	0.00 (17)		

### Hydrogen-bond geometry (Å, °)

**Table:**

*D*—H···*A*	*D*—H	H···*A*	*D*···*A*	*D*—H···*A*
N5—H1···N2^i^	0.88 (1)	2.24 (1)	3.095 (1)	166 (2)
N5—H2···N3^ii^	0.89 (1)	2.15 (1)	3.037 (2)	175 (2)

Symmetry codes: (i) −*x*+3/2, *y*+1/2, −*z*+3/2; (ii) −*x*+3/2, *y*−1/2, −*z*+3/2.
